# Staging prognostic discussions about glioblastoma

**DOI:** 10.1007/s11060-025-05356-8

**Published:** 2026-01-09

**Authors:** John T. Fortunato, Amy Scharf, Andrew G. Shuman, Eli L. Diamond

**Affiliations:** 1 Department of Neurology, University of Michigan, 1500 E Medical Center Drive, Ann Arbor, MI 48109 USA; 2 Center for History, Humanities, Arts, Social Sciences and Ethics in Medicine, University of Michigan Medical School, Ann Arbor, MI USA; 3 Department of Neurology, Memorial Sloan Kettering Cancer Center, New York, NY USA; 4 Ethics Committee and Ethics Consultation Service, Memorial Sloan Kettering Cancer Center, New York, NY USA; 5 Department of Otolaryngology-Head & Neck Surgery, University of Michigan Medical School, Ann Arbor, MI USA

**Keywords:** Glioblastoma, Ethics, Prognosis, Survivorship

## Abstract

**Background:**

Neuro oncologists bear the responsibility of disclosing prognostic information to patients with glioblastoma. Despite this obligation, prognostic information is neither routinely nor effectively communicated.

**Methods:**

A narrative review of empiric data related to prognostic disclosure in cancer and in GBM is performed, and a normative framework based on this data and our own clinical and ethical experience and consideration is presented.

**Analysis:**

The authors propose a framework of staged disclosure of prognostic information, where the incurability of glioblastoma and the likelihood of neurocognitive decline are discussed at the first patient encounter, but estimations of life expectancy are deferred until a subsequent visit. This approach pragmatically balances oncologists’ obligation to preserve patient autonomy and prioritize advance care planning, while also aiming to prevent information overload, allowing the news to be delivered in the context of an increasingly trustful patient-physician relationship, and allowing for more accurate estimations in light of complete pathology results, which are not often available at the first visit.

**Conclusion:**

Staged prognostic discussions about glioblastoma balance oncologists’ ethical obligations and optimize communication of prognostic information to patients and their families. Further empirical studies implementing this approach are warranted.

## Introduction

Glioblastoma (GBM) is an incurable form of brain cancer with a median overall survival (OS) of 15–20 months for patients undergoing standard of care treatment [1, 2]. Despite significant advances in translational and clinical research, no new systemic treatments have been approved for GBM since 2009. After recurrence, prognosis is guarded with a median OS of 8–9 months [3].

Current GBM treatment is directed towards prolonging survival and preserving neurologic function and quality of life (QOL). The current standard of care includes surgery, radiation with concurrent temozolomide, followed by 6–12 cycles of adjuvant temozolomide [1] and/or the use of tumor treating fields (TTF) [4]. Clinical neuro-oncology, in addition to prescribing and guiding treatment decisions, intrinsically involves difficult conversations with patients about individualized goals, treatment preferences, symptom management, QOL, advance care planning (ACP), and end of life care. In the context of GBM patients, these early conversations should address impending cognitive decline, which can worsen throughout the disease course [5] and can leave patients without the capacity to meaningfully make health care decisions, highlighting the need for ACP to occur sooner rather than later. Such discussions are most productive and meaningful when patients are able to acknowledge and understand what their futures likely hold, thereby optimizing their ability to make informed decisions. As such, explicit disclosure by oncologists of prognosis is obligatory for shared medical decision-making.

Presenting individualized prognostic information to a patient with tact and emotional attunement honors their autonomy and is essential to achieving truly informed consent for treatment decisions [6]. The American Society of Clinical Oncology (ASCO) recommends that oncologists discuss prognosis with their patients within one month of any terminal cancer diagnosis [7], prioritizing patient autonomy and informed consent. We propose that the term “prognosis” encompasses two key components: (1) the curability/incurability of one’s disease; and (2) a reasonable estimation of life expectancy, with the caveat that knowledge of these is imperfect. For patients with GBM, a third key component of prognostic information, (3) the likelihood of impending cognitive decline, should be included in early prognostic discussions to afford patients the ability to consider their preferences and participate in decision making prior to their loss of decision-making capacity.

Despite the critical importance of discussing these three components of GBM prognosis to promote informed decision making, there are no relevant neuro-oncology-specific guidelines. While there have been studies amongst general medical oncology regarding the practice of disclosure [8–10], the field of neuro-oncology has a dearth of studies that address the how and when of disclosure. Due in part to extensive provider variation, prognostic information can be inconsistently and ineffectively communicated to patients. A 2014 systematic review of 14 studies on prognostic awareness amongst patients with malignant glioma concluded that there is likely a sizeable portion of patients who are unaware of the incurability of their disease [11]. Surveys have demonstrated that following their neuro-oncology clinic visits, patients often lack a fundamental understanding of their likely disease trajectory and prognosis; more than half were unaware of their life expectancy [12].

In this manuscript, we propose a framework for a staged disclosure of prognostic information for patients with glioblastoma. We recommend that at the first clinic consultation with a patient, neuro oncologists discuss the incurability of GBM and the patient’s likely impending cognitive decline. Discussions regarding estimated life expectancy should ideally wait for a subsequent clinic visit. We recognize that this framework is not static. It should be appropriately and compassionately individualized. Diverse patient characteristics, such as implicit and explicit preferences, as well as clinical estimations of disease trajectory and the timing of pending cognitive decline related to tumor growth, can and should influence the timing and delivery of prognostic information.

In presenting our proposed framework, we first review current trends in clinical practice and perceived barriers to disclosure to explain why prognostic information is often communicated inconsistently or ineffectively. Second, we discuss ethical principles guiding prognostic disclosure as well as patients’ and clinicians’ competing values and priorities that may influence the necessity and timing for disclosing (or not disclosing) prognostic information. Finally, we propose a template sequence for staged prognostic discussions within the timeline of current standard of care therapy for GBM.

## Methods

In this paper, we provide a narrative review of empiric data related to prognostic disclosure in cancer and in GBM, and we propose a normative framework based on this data and our own clinical and ethical experience and consideration. This approach is in alignment with published guidelines for methodology in the writing of medical ethics papers [13, 14].

### Current practice: grappling with hope

Though more common before the 21st century [15], many oncologists still avoid discussing cancer prognosis, particularly early in the disease course. In a 2012 population-based survey of patients with metastatic lung and colorectal cancer, prognosis was first broached roughly one month before death [16]. In a separate study, physicians reported that even if their patients with cancer requested survival estimates, they would provide a frank estimate only 37% of the time [17]. A qualitative study that involved video recording and observing oncologist-patient interactions revealed that “the most frequent way physicians invoke death (i.e., discuss a poor prognosis) is in a persuasive context during treatment recommendation discussions. When patients demonstrate active or passive resistance to a recommendation, physicians invoke the possibility of the patient’s death to push back against this resistance and lobby for treatment.… Ultimately, this study concludes that physicians in these data invoke death to leverage their professional authority for particular treatment outcomes [18],”

When oncologists are asked about their decision to eschew prognostic conversations, they often cite their own fear of destroying their patients’ hopefulness as their central motive [8–10]. Hope is defined in social science literature as “the perceived capability to derive pathways to desired goals and motivate oneself via agency thinking to use those pathways [19].” Tools have been developed to measure hope [20–22], and there are associations between hopefulness and improved physical and mental health [23], as well as patient reported meaning and purpose [24]. Intriguingly, higher levels of hope among patients with advanced cancer are associated with longer survival [25].

Oncologists have a moral obligation to foster hopefulness [26], and workshops have even been created to help us in this endeavor [27]. Callous, insensitive, or ‘tone-deaf’ deliveries of prognostic news can be harmful and damage the therapeutic alliance between providers and patients/caregivers. In one qualitative study, patients with GBM and their caregivers reported their perception of hope being taken away following prognostic discussions with their oncologists. However, further qualitative analysis revealed that physicians had explicitly told patients and caregivers that “there is no hope” or “do you realize that this is hopeless?” [28] In these cases, it may have been the improper delivery and framing of prognostic information by their oncologists, not the prognostic news itself, that reduced hopefulness amongst patients. Though poorly delivered prognostic information might erase hope, research suggests that tactful delivery of prognostic information does not reduce hopefulness. A cohort of 27 patients with advanced cancer showed no change in hopefulness (assessed via the Hope Herth Scale) before and after a prognostic discussion [29]. This finding was later replicated in a much larger cohort of 672 patients with advanced cancer who actually showed a statistically significant increase in hopefulness 3 months after an end of life discussion and ACP [30].

Neuro oncologists’ fear that prognostic disclosure will destroy hope might arise from a narrow interpretation of “hope” as pertaining to only a “hope for a cure.” But, as suggested by Rosenberg and colleagues [31], hope is dynamic and fluid, evolving in complex patterns over time. Patients often have different and even conflicting hopes. Most importantly, these hopes, even if they seem unrealistically optimistic, rarely lead to true misunderstanding [23], but instead serve as psychologically protective mechanisms that “represent exactly what they are – the impossible future that people wish they could have [31].”

The hopes of patients with GBM are likely to change over their course of treatment. Patients may initially hope for a cure. While GBM is almost always incurable, this hope is entirely reasonable and expected. In fact, hope for a cure is shared amongst patients and clinician scientists alike, who are working tirelessly on new treatment paradigms. For neuro-oncologists, this presents an early opportunity to share in their patients’ hopes (for a cure) while simultaneously acknowledging our present clinical reality. Neuro oncologists can widen the concept of attainable hopes for their patients, which may include preserving function for a period, maximizing their perceived QOL, or a ensuring a dignified death. In doing so, the juxtaposition of candor and preserving hope may no longer be at odds. By encouraging patients and caregivers to hold multiple hopes, and over time, to focus on the most realistic of them, neuro-oncologists are meeting patients where they emotionally stand, and may be better-positioned for open discussions on prognosis, treatment options, and ACP.

## Factors favoring earlier prognostic discussion in GBM

### Respect for patient autonomy

In the doctor-patient relationship, respect for patient autonomy is not a passive endeavor. Physicians are required to not only acknowledge a patient’s right to hold views and make independent choices, but to take the required actions that enable and empower patients to do so [32]. Autonomy is a positive obligation, requiring disclosure of medical information so that patients can achieve a level of understanding that allows them to make autonomous choices. Particularly in oncology, this requires disclosure of prognostic information in addition to medical information. Such disclosure, or truth-telling, by oncologists is far easier when the prognosis is favorable and will not cause angst for the patient or their caregivers. For example, when a pathology result is promising, clinicians (anecdotally) often willingly and without hesitation reveal all known prognostic information to their patient. Physicians are no less ethically obligated to respect patient autonomy and disclose a poor prognosis, although this is far more challenging and requires tact, thoughtfulness, compassion, and time.

### Impending cognitive decline and early advance care planning

ACP and prognostic discussions should be held early in the GBM disease course due to the possibility of early cognitive decline and progressive disease in a compressed timeline [33–37]. At initial diagnosis, approximately half of patients with high grade gliomas have diminished medical decision making capacity, which are likely attributable to disease-related cognitive deficits [38, 39]. GBM patients’ cognition can worsen throughout the disease course [5] and often declines rapidly near the end of life [40]. Even when cognition is well preserved, mental status can fluctuate due to various dynamic processes, such as seizures or infection, resulting in a temporary loss of decision-making capacity.

ACP between physicians, patients, and caregivers involves appointing a health care agent, making decisions regarding treatment and symptom management, and clarifying end-of-life preferences. Because patients with GBM are at significant risk for cognitive decline, it is necessary to respect their autonomy by having prognostic conversations to ascertain (and document) their stated wishes prior to a loss of capacity that precludes them from meaningfully participating in their care. Caregivers are often responsible for making medical decisions for GBM patients near the end of life, and they should be equipped to make decisions that are informed by, and concordant with, the patient’s preferences. ACP discussions early in the disease course empower patients to articulate their wishes and treatment goals and ease the decision-making burden on family members (or other surrogate decision makers). Involvement of next of kin or other caregivers in the ACP process from the outset can also provide additional support for the patient, and can facilitate more robust ACP conversations [41]. Patient advocacy organizations often offer patient-focused materials and other resources that can enrich ACP and can provide counseling regarding financial toxicity. Finally, palliative care providers are often able offer expertise in assisting with ACP that can benefit patients, particularly when offered early in the disease course [42].

Though it is generally accepted that “early” ACP is preferred, the precise timing is not clear. Everyone should appoint a health care agent who can make medical decisions on their behalf in the event they lose capacity. While cognitive decline may be present at initial diagnosis and can worsen drastically, its overall trajectory is unpredictable and can be influenced by several factors (ex: tumor location, use of steroids, seizures, and use of antiseizure medicine). As such, we recommend that prognostic discussions ought to precede further ACP, as the impetus and urgency for early ACP is directly related to the projected clinical course. If ACP ought to be early, prognostic disclosure should occur even earlier.

## Factors favoring later prognostic discussion in GBM

Despite the preference for early prognostic discussions, there are multiple clinical, psychosocial, and ethical considerations that might favor a deliberate deferral of prognostic disclosure. When considered together, the following factors suggest that thoughtfully deferring the delivery of some prognostic information to patients with GBM might be optimal (Table 1).

**Table 1:** Reasons why neuro-oncologists might have prognostic discussions with GBM patients at the first clinic visit vs. subsequent clinic visits

Early disclosure:	Deferred Disclosure:
Autonomy	Information overload
Pending cognitive decline	Relationship building
Early Advance Care Planning	Delayed results of tumor genetic testing and MGMT status

### Information overload

Recall of clinical information amongst all cancer patients is poor [43–45]. Patients with cancer do not retain 40–80% of information given to them by healthcare providers [46–49]. Further, it has been demonstrated that patients with a poor prognosis have less recall, and the more extensively one’s prognosis is discussed, the less information is recalled [46]. Patients with GBM are similarly vulnerable to poor recall of prognostic information, particularly at their first meeting with their neuro oncologist. In a qualitative analysis of 21 patients with HGG and 19 caregivers, 100% of patients reported a sense of “shock” after hearing their diagnosis, and “trying to understand and process prognostic information while still in shock” was a common theme amongst many participants. Some participants could not recall the details of their prognosis afterward [28]. At their initial consultations with neuro-oncologists, patients usually have other appropriate and pressing questions (i.e., what is a GBM? What are the treatment options? My surgeon told me we “got all of it” – why do I need more treatment? ) Oncologists often spend an hour or more introducing the disease and discussing future treatment plans, and there are valid concerns that adding a comprehensive prognostic conversation to this first consultation might be overwhelming for patients and caregivers.

### Relationship building

We theorize that patients may be more receptive of some prognostic information once their relationship with their oncologist has been more firmly established. Anecdotally, we have observed that some GBM patients, when obtaining a second opinion, recall their primary neuro oncologist’s prognostic discussions to be off-putting and even distrustful. Even when the primary neuro oncologist’s prognostic information was accurate, patients considered them to be strangers delivering devastating news.

Patients who report longer relationships with their physicians are more likely to trust their physician [50], and this trust is associated with greater satisfaction with their physicians’ communication [51, 52]. Relatedly, cancer patients are more likely to be adherent to treatment recommendations when they trust their oncologist [52, 53]. Therefore, there may be value in a deferred delivery of some prognostic information until later, once a trusting patient-physician relationship has formed.

### Time frame for results of tumor genetic testing

The 2021 WHO classification of tumors of the central nervous system (CNS) introduced major changes that highlight the now-prominent role of molecular diagnostics in CNS tumor classification [54]. Treatment informed by advanced molecular testing (next generation sequencing [NGS]) can impact survival for patients with advanced cancer [55], and NGS is now a routine part of clinical oncology practice, including neuro-oncology. Other molecular biomarkers, such as O6-Methylguanine-DNA Methyltransferase (MGMT) profiling, are not included in NGS panels, but are standards of care and have both therapeutic and prognostic implications [56]. Prognostic estimations of life expectancy can be more precise once these data (particularly MGMT methylation status) are known, and these advanced tests can take up to 4–6 weeks to be processed. Many GBM patients first meet their neuro oncologists within 1–3 weeks after a diagnostic craniotomy, meaning that these prognostic data remain pending, thereby precluding the most prognostically accurate discussion. This consideration constitutes an additional legitimate rationale for deferring prognostic discussion beyond earliest visits in many cases.

## Balancing obligations via staged disclosure

Neuro-oncologists often do not provide patients with the initial disclosure of a brain tumor diagnosis because of the typical clinical presentation and diagnostic trajectory, but they bear the responsibility of expanding on the diagnosis, treatment, and its prognosis to patients and caregivers. Establishing with a neuro oncologist after diagnosis can sometimes take several weeks, and when oncologists and patients hold their first consultation, the patient and caregivers have sometimes already received some diagnostic and prognostic information from other providers. However, this information is often incomplete or piecemeal, in part because other teams appropriately defer to the oncologist, and histological and molecular reports have not been finalized. Some patients may be under the impression that their neurosurgeon has removed their glioma in its entirety, believing that they have been cured. Additionally, many patients have conducted their own research about glioblastoma on the internet.

Given this context, we recommend that at the first clinic visit, oncologists explicitly disclose and discuss the incurability of GBM. Proactively framing a glioblastoma diagnosis as an incurable disease mitigates the risk of potential misalignment of patient expectations with expected clinical outcomes. Further, it allows treatment discussions, which are logistically essential at this first oncology visit, to be contextualized as aiming to prolong life and optimize QOL within a rubric of incurability. This understanding is necessary for patients to be able to provide informed consent for subsequent treatments and reduces the chance for patients to misconstrue treatment intent as curative.

Second, we recommend that at the first clinic visit, oncologists explicitly disclose the likelihood of cognitive decline associated with GBM for all patients. Some patients may initially present with excellent functional status and without baseline cognitive deficits, while for others, cognitive impairment may already be incipient. Projected cognitive decline may be more evident for certain patients based on tumor location, but some form of cognitive decline can be expected for the majority patients due to tumor progression, radiation sequelae, anti-seizure medications, steroids, or a conglomeration thereof [5, 40]. This disclosure allows the oncologist to highlight the relevance of formally appointing a next of kin, family member, friend, or other individual as the health care agent who invariably will become the primary decision maker for the patient at some point [38, 39]. In addition, this disclosure illuminates the importance of early ACP, which includes encouraging the patient to expeditiously and formally engage with their caregivers, express their preferences for end of life care, and organize personal affairs.

For most patients, confirmation of their diagnosis of glioblastoma and the news of its (1) incurability and (2) its attendant cognitive decline will be overwhelming, particularly if they had been under the impression that their brain tumor was curable or indeed cured by surgery. Due to the breadth of other clinical information that must be conveyed at this first visit (diagnostic information, medication management, treatment logistics including the potential use of TTF), we recommend oncologists refrain from offering estimations of life expectancy (unless they are explicitly requested, see “Special Circumstances” below). This deferral may lessen information overload and allow the physician to build rapport and garner trust with the patient and caregivers. Further, oncologists can offer greater prognostic accuracy later, when molecular testing (MGMT testing) has been completed. Oncologists may choose to acknowledge the gravity of information provided and solicit the patient’s willingness to hear more, openly highlighting their attempt to balance the competing principles stated above. Patients should be told that they can reach out to the oncology team via patient portal for follow up questions. If needed, a short interval follow-up phone call with an oncology nurse a few days after the clinic visit can be offered to help mitigate information overload and answer remaining questions. Early palliative care referrals should be considered, particularly if patients have uncontrolled symptoms or other QOL related issues [42].

**Fig. 1:**
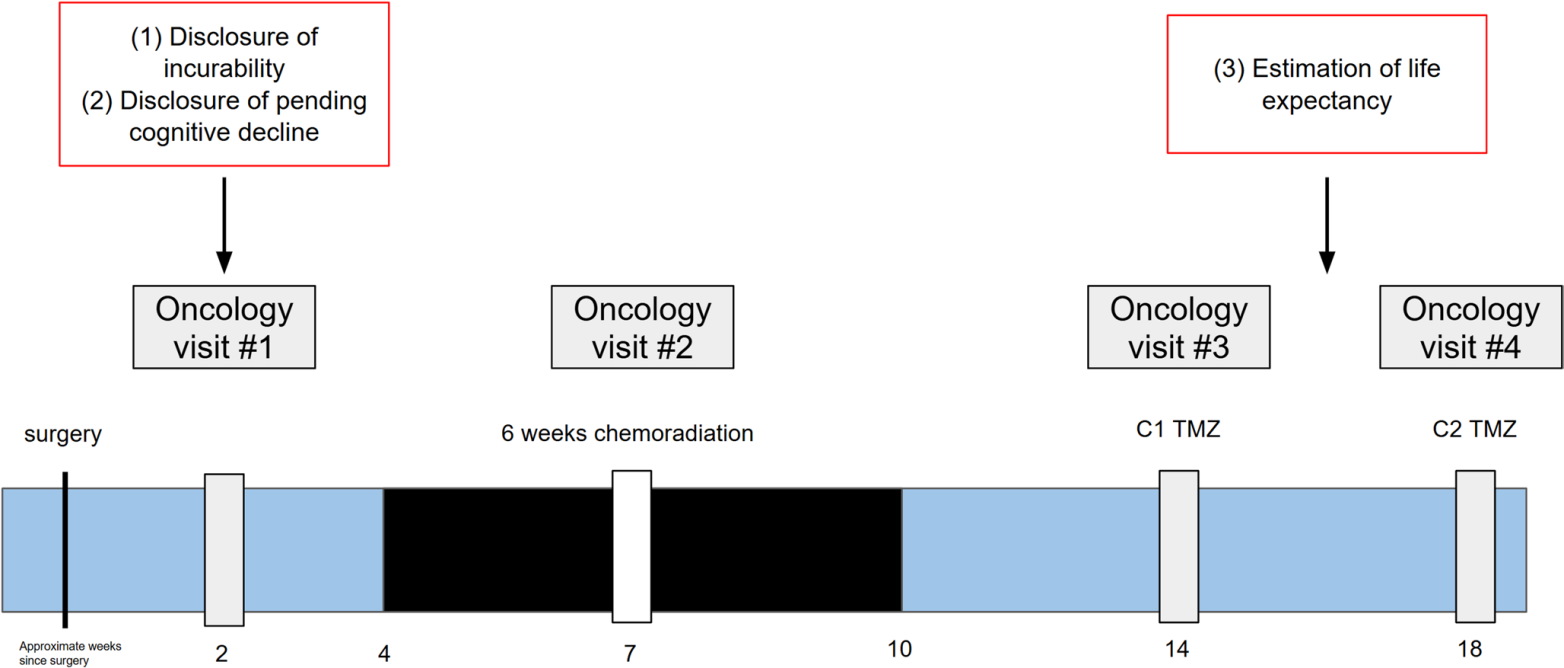
Timeline of staged disclosure of prognostic information for patients with glioblastoma

Throughout subsequent clinic visits, when more prognostic information is available and the physician-patient relationship has been further solidified, we recommend that oncologists complete the next stage of prognostic disclosure and explicitly discuss life expectancy (Fig. 1). Typically, patients return to see their oncologist one month after completing radiation, and then again 4 weeks later after their first cycle of chemotherapy – a total period of approximately 2–3 months following their initial diagnosis. This timing for discussions about life expectancy may be appropriate because patients will have developed a relationship with their oncologist, who can tailor prognostic information based on observed and expressed patient-specific preferences. Also, molecular diagnostic data, including MGMT testing, should be completed, providing oncologists with greater accuracy in prognostic estimations. Ongoing treatment plans (moving onto adjuvant chemotherapy after radiochemotherapy) rarely change at this juncture. This affords the oncologist, patient, and caregiver more time for thoughtful prognostic discussions, such as detailed estimations of life expectancy, without having to navigate new treatment logistics.

While it is common for oncologists to cite median overall survival data during these conversations, it should be acknowledged that patients can have difficulty contextualizing statistical information. In a study of women with breast cancer, 73% of patient did not understand the term “median survival” [57]. In our practice, we often explain median overall survival data by stating that “half of the patients studied lived longer, while half of the patients studied lived shorter than ‘X’ months.” Patients should be told that statistics are averages across the population and inherently are not tightly applicable for any given individual, but instead are meant to help guide decisions and discussions.

Staged disclosure allows us to balance competing priorities by first discussing the incurability of GBM and expected cognitive decline, prioritizing autonomy, and allowing for early ACP before cognitive impairment worsens, but thoughtfully deferring discussions of life expectancy in favor of avoiding information overload. This allows for the most sensitive news (estimations of life expectancy) to be delivered in the context of an established and trusting oncologist-patient relationship, and for accurate estimations in light of molecular results and MGMT testing.

One potential objection of staged disclosure is that withholding this information is paternalistic and limits patient autonomy. We posit that thoughtful timing of the delivery of a life expectancy estimation actually promotes patient autonomy, as it allows patients to make informed decisions based on the most relevant and complete information available. The staged method does not delay the initiation of ACP, a bedrock of patient autonomy. Patients should be encouraged to consider ACP from their initial diagnosis, with the acknowledgement that their wishes and preferences may change over time and over the course of their disease. Discussing life expectancy later, when all diagnostic test results are confirmed, patients’ relationship with their oncologist is established, and when there is less risk of information overload, may actually lead to patients having a greater understanding to inform their decisions. Finally, in our proposed staged process, the notion of incurability is discussed in earlier stages, which is fundamental to autonomous decisions about initial treatment. Some patients may request to initiate discussions about life expectancy at their initial visits. Doing so in such instances is a legitimate affirmation of autonomy.

An alternative critique of a staged disclosure approach might be that for the sickest patients, withholding discussions about life expectancy carries an unacceptable risk of harm. We maintain that for the rare patients with rapidly progressive disease during chemoradiation, earlier prognostic conversations would have, in hindsight, been inaccurate anyway. However, for the sickest patients, such as the elderly, those with tumors undergoing biopsy only, or those with poor baseline functional status, we agree that disclosure of estimations of life expectancy are often necessary sooner and cannot be delayed – particularly in situations where foregoing any cancer directed treatment might be considered. Staged disclosure offers a template from which oncologists can approach many patients, from which adjustments can be made based on careful consideration of competing values and priorities. Staged disclosure is not prescriptive nor one-size-fits-all and should be tailored for each patient individually.

## Special circumstances

### Explicit request for early survival-related information

If the patient asks for an estimate of life expectancy at the first clinical encounter, the clinician has an obligation to pivot from the staged disclosure model and provide requested information as accurately and completely as possible, following established frameworks for responses to this question [58]. It is reasonable for the clinician to elucidate on the current unknowns in providing an accurate answer, and then map out expectations based on different potential scenarios (ex. If your tumor is MGMT methylated or unmethylated, this means…). In such an instance, the oncologist could consider giving the patient the opportunity to reconsider their request for survival statistics in that moment, by communicating that some patients find this information difficult to absorb in the setting of a first visit, leading them to feel overwhelmed. Nonetheless, the oncologist will have that candid discussion any time it is desired. It should also be acknowledged that many patients or caregivers will have already read about survival data via the internet or with the assistance of artificial intelligence. While some internet resources can be accurate, others are not. Patients should be encouraged to appraise these resources in partnership with their oncologist. A clear request for survival information should certainly be met with candor and compassion.

### Clinical trial enrollment

For patients enrolling onto clinical trials for newly diagnosed GBM, we recommend that the disclosure of both incurability and life expectancy be completed before enrollment. To provide truly informed consent for experimental therapeutic trials, patients must first understand the modest efficacy and expected outcomes from standard of care treatments. Though *therapeutic optimism* (hope for a meaningful clinical benefit from a therapeutic clinical trial) is common among patients, clinicians and researchers should monitor and rectify *therapeutic misconception* (conflation of the aims of clinical research and the belief that there is direct benefit fundamentally from clinical trial enrollment) [32, 59].

### Concerns for extreme distress or suicidality

There are infrequent instances when physicians suspect that providing prognostic information, such as the incurability and diminished life expectancy of a GBM diagnosis, might be so distressing to a patient that it could cause suicidality, or interfere with their capacity to make healthcare decisions, and/or drastically reduce their QOL. Only in these rare circumstances, where potential patient distress from prognostic insight might limit their decision making capacity (and thus actually limit patient autonomy) is the nondisclosure of prognostic information, often referred to as therapeutic privilege, morally permissible [60, 61]. Close follow up and monitoring of these patients through frequent clinic visits is recommended. Family support, when appropriate, as well as institutional support (psychiatric care, social work, patient advocacy) should be offered. Ethics consultation and support from the ethics committee might be beneficial to the oncologist in these challenging situations. Future efforts to disclose prognostic information should be made, as soon as it is determined to align with the patient’s best interests and no longer limits their autonomy.

### Patient’s right to decline prognostic information

Some patients might explicitly request that the physician withhold prognostic information. Often, these patients make statements like “I only want to hear the good news” or “I just need to stay positive at this time.” Oncologists might be uncomfortable with these requests, as it is difficult for patients to make informed decisions about their care without an accurate understanding of their prognosis. However, just as patients have a right to receive information about their health, they also possess the same right to decide to not be informed. The ethical principle of relational autonomy supports a patient’s right to appoint a family member or loved one to receive prognostic information and make informed decisions on their behalf. Patients with decisional capacity have the right to make such choices in accordance with their personal preferences and values, and clinicians have an ethical responsibility to respect these choices. However, patients should be periodically asked, particularly at important junctures in their care, to re-affirm their preferences.

### Requests by caregivers to withhold information to patients

Some individuals, families, and communities have different perspectives with respect to truth-telling, and in some cases family members may ask or even demand that oncologists not reveal to patients their diagnoses or prognoses. There may be strongly held cultural and/or religious beliefs that it is the family’s responsibility to receive information and make decisions based on what they feel is in the patient’s best interest, and that a physician’s disclosing medical information directly to the patient is not acceptable. Both legally and ethically, all patients have the right to receive information regarding their medical status and care, which enables them to make choices consistent with their goals and values, including delegating information disclosure and/or decision making to others. When confronted with requests by caregivers to withhold or circumvent disclosure to patients, physicians should state a clear respect for familial and cultural values. At the same time, physicians should explain to caregivers that out of respect for patient autonomy, the medical team will not lie to patients. It is often possible to confirm that the patient shares these familial or cultural values by asking them whether they prefer to receive information about their condition directly or whether they prefer that family members be the primary contact for information and decisions. Physicians are obligated to respect each patient’s right to decide the extent to which they want to be involved in their care, and that they will attempt to ascertain from them– in a sensitive and compassionate way -- what their wishes are regarding information sharing and decision-making.

### Special considerations for young adults

It is beyond the purview of this project to delve into prognostic discussion practices in the setting of children and adolescents with brain cancer. While 18 is considered the age of consent, young adults may not be emotionally or cognitively ready for comprehensive discussions of prognosis as outlined in our staged process. For the years of early adulthood, oncologists must thoughtfully involve parents and guardians in the process of prognostic discussion while maintaining respect for the patient’s autonomy and privacy. This is a challenging process in the young adult population, combining aspects of adult and pediatric disclosure practices, which likely merits independent consideration.

## Conclusion

We propose a staged disclosure of prognostic information for patients with newly diagnosed glioblastoma; first with the disclosure of the incurability of GBM shortly after diagnosis, then 1–2 months later with a discussion of life expectancy. Staged disclosure is ethically justified, as it allows the patient and physician to develop a trusting relationship, aims to prevent information overload, and fosters hope without limiting autonomy or interfering with ACP. This approach prioritizes patient respect by tailoring disclosure based on ethical principles and patient-specific and practical/logistical factors. Further empirical studies implementing this approach and then assessing the prognostic awareness of patients should be used to validate this framework.

Via staged disclosure, prognostic discussions can appropriately take place over several months. Thus, broader ASCO guidelines for cancer prognosis disclosure may not neatly apply to GBM. Given the disease-specific needs of patients with brain cancer, we recommend that the Society of Neuro-Oncology consider forming a collaborative working group, including clinicians, patients and caregivers to create formalized guidelines for prognostic disclosure.

A formalized framework with staged disclosure provides a roadmap for neuro oncologists to approach clinical encounters. It should increase prognostic awareness amongst patients, allow treatment and end of life care to be more in alignment with patient wishes, and may lead to decreased use of unnecessary medical interventions at the end of life. As new diagnostic tests are developed and new treatments emerge, these practical considerations might change. But our obligation to balance truth telling, preservation of hope, respect for patient autonomy, and relationship building will remain paramount.

## Data Availability

No datasets were generated or analysed during the current study.
